# Phosphorus Fertilizers from Sewage Sludge Ash and Animal Blood as an Example of Biobased Environment-Friendly Agrochemicals: Findings from Field Experiments

**DOI:** 10.3390/molecules27092769

**Published:** 2022-04-26

**Authors:** Magdalena Jastrzębska, Marta K. Kostrzewska, Agnieszka Saeid

**Affiliations:** 1 Department of Agroecosystems and Horticulture, Faculty of Agriculture and Forestry, University of Warmia and Mazury in Olsztyn, Plac Łódzki 3, 10-718 Olsztyn, Poland; marta.kostrzewska@uwm.edu.pl; 2 Department of Engineering and Technology of Chemical Processes, Faculty of Chemistry, Wroclaw University of Science and Technology, Wyspiańskiego 42, 50-376 Wrocław, Poland; agnieszka.saeid@pwr.edu.pl

**Keywords:** phosphorus recycling, microbial solubilization, recycled fertilizers, *Triticum aestivum* ssp. *vulgare*, grain yield, soil properties, potentially toxic elements

## Abstract

Wastes of biological origin from wastewater treatment systems and slaughterhouses contain substantial amounts of phosphorus (P) with high recovery potential and can contribute to alleviating the global P supply problem. This paper presents the performance of fertilizer (AF) and biofertilizer (BF) from sewage sludge ash and animal blood under field conditions. BF is AF incorporated with lyophilized cells of P-solubilizing bacteria, *Bacillus megaterium*. In the experiments with spring or winter wheat, the biobased fertilizers were compared to commercial P fertilizer, superphosphate (SP). No P fertilization provided an additional reference. Fertilizer effects on wheat productivity and on selected properties of soil were studied. BF showed the same yield-forming efficiency as SP, and under poorer habitat conditions, performed slightly better than AF in increasing yield and soil available P. Biobased fertilizers applied at the P rate up to 35.2 kg ha^–1^ did not affect the soil pH, did not increase As, Cd, Cr, Ni, and Pb content, and did not alter the abundance of heterotrophic bacteria and fungi in the soil. The findings indicate that biobased fertilizers could at least partially replace conventional P fertilizers. Research into strain selection and the proportion of P-solubilizing microorganisms introduced into fertilizers should be continued.

## 1. Introduction

According to current estimates, the number of people in the world is increasing by more than 200,000 individuals every day [1]. If growth tendencies continue, the world population will approach 9 billion by 2037 and reach 10 billion in 2057 [1]. Since agriculture nowadays provides ~97.0% of the global food supply [2], it is mainly this sector that will face the dilemma of feeding the world in the coming decades [3]. The required corresponding increase in food production must come from increased crop productivity (higher yields, cropping intensities) [4,5] because the expansion of agricultural land is limited [4]. An additional challenge is to increase agricultural productivity on a sustainable basis, i.e., without impacting environmental security, while conserving and enhancing biotic and abiotic resources [3,6]. Reducing food loss and waste should be an accompanying strategy [7].

Increased crop productivity comes with the need for plants to take up more nutrients, mainly from soil resources. Soil nutrient abundance or nutrient availability often does not meet the nutritional requirements of high production potential crops, and replenishment of nutrients from external sources is necessary [8]. Chemical/mineral fertilizers are fast-acting nutrient carriers that promote plant growth by rapidly increasing soil fertility [9].

Current world agriculture consumes 107.7, 43.4, and 37.4 million tons of N, P_2_O_5_, and K_2_O, respectively and, on average, per 1 hectare of cropland, 69.2 kg N, 27.9 kg P_2_O_5_ and 24.0 kg K_2_O are applied in the mineral fertilizers [2]. However, the global use of fertilizers is highly unbalanced and both overfertilization and fertilizer underutilization occur in different world parts. [10]. Both fertilizer underuse and overuse cause specific problems in the regions of occurrence [11,12]; however, in recent years, more attention has been paid to the consequences of the latter [13,14,15].

The adverse effects of mineral fertilizers on human health (including the formation of carcinogenic nitrosamines in the human body, methemoglobinemia, health risks of heavy metals, ‘hidden hunger’) and the environment (including soil acidification, toxic element pollution, eutrophication of aquatic ecosystems, global warming, alterations of biotic communities) have been addressed in numerous publications [16,17,18,19]. The production of mineral fertilizers requires high energy input [20]. What is more, they are based on fossil fuels (N-fertilizers on Haber–Bosch process) or fossil ore deposits (phosphate rock), which are finite, non-renewable resources [21].

In the past decade, the concept of the so-called circular economy (CE) has gained momentum [22]. Regardless of its many definitions [23], it generally implies closing material loops to preserve products, parts, and materials in the industrial system and extract their maximum utility [24]. CE is seen as a promising strategy for supporting sustainable agriculture [25].

One of the practical approaches towards CE is nutrient recovery from biological waste and their application in the form of biobased fertilizers [26]. Some materials of biological origin (biomass) are traditionally used directly as organic/natural fertilizers or soil improvers, e.g., animal manure and crop residues. In addition to these so-called agricultural wastes, the organic fertilizer industry currently recycles organic by-products from various industries (value chain), including food and beverage, forestry, wood, paper and packaging, cosmetics and pharmaceuticals, environmental management, petroleum, and textiles [27]. Among waste streams, animal manure, sewage waste and food chain waste, especially slaughterhouse waste, are considered the most promising substrates for fertilizer production [28]. Most wastes require further processing for various reasons and, depending on the substrate, different technologies are recommended, including drying, composting, biological treatment, anaerobic digestion, incineration, liming, NP-precipitation, among others. As a result, various types of organic, organic-mineral, and inorganic fertilizers from renewable sources are developed [27]. Despite occurring estimates, the empirical knowledge on the markets for waste and waste-derived fertilizers used in agriculture is still insufficient [29]. The content of fertilizer components, however, estimated at about 22 million tons/year for nitrogen and 1.3 million tons/year for phosphorus, argues for the huge potential of biomass waste streams [27].

The case of phosphorus (P) recycling and the real possibility of replacing or supplementing phosphate rock-based fertilizers with recycled ones show that CE principles work in practice [30,31]. Phosphate rock, the main raw material base for phosphate fertilizers, is a limited, non-renewable resource and, in addition, unevenly distributed around the globe [32]. The scarcity of phosphate rock is a serious problem for Europe, making it dependent on importing virtually the entire raw material needed. In 2014, phosphate rock was placed by the European Union (EU) on the list of critical raw materials, to which P was also added in 2017 [33,34]. For these reasons, many European countries have intensified efforts to use renewable secondary P resources more efficiently [35]. Abundant P-rich waste streams of biological origin flow from municipal and industrial wastewater treatment systems and slaughterhouses [36], and this reservoir can be recovered through different technologies and be re-used in the form of new generation fertilizers [27,36]. In recent years, some countries have begun to develop legislative means to enforce P removal from wastewater and P recovery, and Switzerland was the first country to make P recovery from sewage sludge and slaughterhouse waste mandatory [37]. From July 2022, a new regulation of fertilizer products will come into force in the EU to promote the use of fertilizers made from organic or recycled materials [38].

Given the growing problems associated with sewage sludge disposal (growing production [39], emerging new chemical and biological contaminants [40], legal restrictions for agricultural use [41]), thermal sludge conversion is thought one of the most promising methods of sludge management [42]. Raw sewage sludge ash (SSA), while classified as waste [43], is also seen as a potentially valuable source of P for fertilizer production [44,45]. According to common recent estimates, the annual global production of SSA is about 1.7 million tones and is expected to increase in the future [46]. The P content in dry matter of SSA ranges from less than 10% to less than 20% [47], which is comparable to the content of this element in commercial phosphate rock (10.9–16.13% P) [48]. Apart from P, SSA is also a carrier of other macro-and micronutrients [49]. Although the potential of P recovery from SSA is high, the P bioavailability in SSA is low and more than half of the ashes cannot be used as fertilizers due to their high potentially toxic element (PTE) content [44]. In recent years, several new technologies have been developed with the potential to convert SSA into marketable fertilizer products after further treatment [44,45].

Animal blood is considered one of the main by-products of slaughterhouses, which, after being dried and powdered [50], is widely used as blood meal, an environment-friendly fertilizer [51]. Although blood meal is not rich in P (only 0.22% according to [52]), it contains a high proportion of N (about 12% N [52]) in the N-NO_3_ form, which is readily available for plants [53], and trace elements [52]. In addition, animal blood is a good binder that can be used in fertilizer production [52].

A new, innovative approach in waste-based fertilizer production is manufacturing these agrochemicals using a microbial solubilization process [54]. Many naturally occurring microbial organisms, including bacteria, fungi, actinomycetes and algae, exhibit P solubilization ability. Among all microorganisms in the soil, phosphorus-solubilizing microorganisms (PSM) can constitute up to 50% [55]. They are mostly associated with the plant rhizosphere. The most powerful PSM are strains from the bacterial genera *Pseudomonas*, *Bacillus*, *Rhizobium* and *Enterobacter* along with *Penicillium* and *Aspergillus* fungi, and *Bacillus megaterium* is reported as one of the most important strains [55]. PSM solubilize inorganic P compounds via the release of organic and inorganic acids and phosphatase enzyme [56]. In addition to P solubilization, other biological mechanisms involving PSM (e.g., nitrogen fixation, potassium solubilization, phytohormone excretion, the release of antibiotics and antifungal metabolites, guarding plants from abiotic and biotic stresses and pollutant detoxification) function in soil, allowing them to promote plant growth directly or indirectly [57,58]. PSM have already been used in agriculture: alone, to activate ‘legacy P’ [59], or with P substrate of low plant availability [60]. Any substance containing live microorganisms that exhibit beneficial properties for plant growth and development is named a biofertilizer [61]. Recently, PSM have also been shown to effectively solubilize P from waste materials [62]. These findings provided the basis for the development of a technology to produce biofertilizers from secondary raw materials in a formulation in which living PSM cultures were incorporated [54].

The recommendation of all waste biomass-based agrochemicals as substitutes for conventional fertilizers should be based on the results of their agronomic evaluation conducted under real (field) conditions. This evaluation should include not only a yield-forming efficiency analysis but also agricultural product quality and environmental impact. Although many papers on modern P recovery technologies and recycled fertilizer production have been published in recent years [44,45,63,64,65,66,67], field test reports of such agrochemicals are not very common [68,69].

This paper presents the major results of the field evaluation of two biomass-based agrochemicals produced from SSA and dried animal blood, i.e., fertilizer (AF) and biofertilizer (BF, i.e., AF containing *B. megaterium* cells). The aim of the study was to assess the effect of these chemicals on wheat (test crop) productivity, i.e., grain yield and yield structure components, and on selected properties of the soil environment under the test crop, i.e., pH, available P content, the content of PTE (As, Cd, Cr, Ni and Pb) and the abundance of heterotrophic bacteria and fungi. It was hypothesized that the new biomass-based fertilizers would not show inferior yield-forming efficiency versus a commercial P fertilizer, superphosphate (SP), and their impact on the soil environment should be the same or more beneficial than that of SP.

## 2. Materials and Methods

### 2.1. Fertilizer Characteristics

In field conditions, two biomass-based P agrochemicals were applied: a biomass-based fertilizer (AF) and a biomass-based biofertilizer (BF). The raw materials for their production were: ash from the incineration of sewage sludge biomass from wastewater treatment (sewage sludge ash, SSA; Olsztyn, Poland) and dried animal (porcine) blood. The BF was additionally incorporated with lyophilized cells of *Bacillus megaterium*, a P-solubilizing bacteria strain. The SSA was supplied by the Municipal Wastewater Treatment Plant ‘Łyna’ in Olsztyn (Olsztyn, Poland). Dried blood was sourced from the meat processing industry. Bacterial lyophilizate was acquired from the Polish Collection of Microorganisms at the Institute of Immunology and Experimental Therapy of the Polish Academy of Sciences in Wrocław (Wrocław, Poland). Application batches of AF and BF as granules were prepared at the Institute of New Chemical Syntheses in Puławy (Puławy, Poland) [70] following a concept elaborated at the Wrocław University of Science and Technology (Wrocław, Poland) [54]. Table 1 shows the elemental composition of the biobased fertilizers under study.

Superphosphate Fosdar^TM^40 (Grupa Azoty FOSFORY Sp. z o.o., Gdańsk, Poland), purchased on the market, was used as the reference fertilizer in the study. According to the fertilizer label information, it contained 17.6% P, 7.15% Ca, 2.00% S and microelements (B, Co, Cu, Fe, Mn, Mo, and Zn).

### 2.2. Experimental Design and Agronomic Management

Three field experiments were conducted with common wheat (*Triticum aestivum* ssp. *vulgare* MacKey), spring (SW-2016, SW-2017) or winter (WW-2017; sown in 2016), as a test plant. In each experiment, AF and BF were tested against SP and no P treatments (P_0_). All P-fertilizers were applied at three different P doses: (1) 17.6, (2) 26.4, and (3) 35.2 kg ha^–1^. As a result, ten treatments of P fertilization were compared (Table 2; [71]).

P-fertilizer application on experimental plots was performed pre-sowing, simultaneously with potassium (K) fertilizers and the first part of the nitrogen (N) fertilizer dose. The fertilizers were distributed manually on the plot surface and were then mixed with soil by harrowing. Detailed agronomic information is provided in Table S1.

The experiments were arranged in a randomized block design. In each experiment, individual experimental treatments were executed in four replications (plots of 20 m^2^ each).

### 2.3. Study Site, Soil and Weather Conditions

The study area was located in north-eastern Poland (Warmińsko-Mazurskie Province), in the fields of the Production and Experimental Station ‘Bałcyny’ Sp. z o.o. in Bałcyny (53°35′46.4″ N, 19°51′19.5″ E; 136.9 m above sea level). A temperate climate and glacial landform characterize the region. In Bałcyny, soils of the Luvisols type are the most common. Each experiment was established on a soil that satisfied the species requirements of the test plant. Basic soil characteristics prior to the start of each experiment are shown in Table 3. Before the start of the SW-2016 experiment, the soil was also tested for PTE total content. It contained, on average: 1.56 (max 5.655) mg kg^–1^ As, 0.140 (max 0.687) mg kg^–1^ Cd, 22.4 (max 26.3) mg kg^–1^ Cr, 8.94 (max 20.54) mg kg^–1^ Ni, and 1.67 (max 9.90) mg kg^–1^ Pb.

The meteorological conditions in the period of the study are presented in Table S2. The weather conditions during the 2016 growing season were favorable for spring wheat development. Warm weather and spring rainfall (late April and early May 2016) resulted in even plant emergence. Further intensive wheat development from tillering to heading continued under favorable moisture and thermal conditions (May, June 2016). During the setting and filling of grain, abundant precipitation was observed, but its uniform distribution in time had a beneficial effect on reserve accumulation.

Excessive rainfall at the end of spring wheat vegetation (July 2016) delayed grain maturation. The average monthly air temperatures during the 2016/17 growing season did not differ strongly from the averages established for 1981–2010, while the precipitation distribution differed slightly from that typical for the region. October and November 2016 were slightly cooler than expected, with increased precipitation. December 2016 was relatively warm and rainy. The cooling in the autumn months came gradually, which contributed to the proper winter wheat crop preparation for overwintering. In January 2017, the air temperatures were lower than on average for 1981–2010, and the precipitation was scarce.

The snow cover only increased in February. Winter wheat did not experience the negative effects of the winter season. March was already warm and rainy. April also precipitated abundantly, although it was cooler than usual. These conditions delayed the sowing of spring wheat and then its emergence. A slightly drier May 2017 was followed by rainy periods in June and July and the beginning of August. Such conditions resulted in prolonged ripening and made harvesting the crop on time difficult. Weather conditions, by regulating the soil water content, can determine the availability of P and other elements to plants [73], influence the activity of microorganisms introduced with the biofertilizer [74], and also affect the biological soil activity [75]. Abundant rainfalls can promote the leaching of nutrients and PTE from the topsoil into groundwater or aquatic ecosystems [76].

### 2.4. Sampling, Measurements and Analyses

#### 2.4.1. Grain Yield and Yield Structure Components

The wheat grain yield was evaluated in each of three experiments, based on the amount harvested from individual experimental plots, with the results calculated for 1 ha area and grain moisture of 12%. The wheat yield structure parameters were determined as follows: spike density per 1 m^2^ was counted with the frame method prior to wheat harvest, grain number per spike was based on measurements on 25 wheat plants sampled from each plot before harvest (spikes threshed and grains counted manually), and 1000 grain weight (TGW) was derived based on grain samples (about 1 kg) collected during the harvest.

#### 2.4.2. Soil pH

In all experiments, soil pH was investigated on three dates: (1) before the start of the experiment; (2) at the wheat tillering stage (winter wheat has reached this stage in spring); (3) after wheat harvest. Soil samples were taken from the 0–30 cm soil layer using a hand-held twisting probe (Egner’s soil sampler). Soil material was collected from each plot separately, from 30 points distributed evenly throughout the plot surface, thereby gaining a sample of approximately 1 kg from a single plot. The collected soil material was dried at room temperature for several days and then thoroughly mixed and sieved. Afterwards, the separated 300 g portions were submitted to the Chemical and Agricultural Station in Olsztyn (Poland), where the pH in 1 M KCl was assayed by potentiometry [77].

#### 2.4.3. Total Element Content in Soil

The total content of C, N, P, K, and Mg was determined in three experiments before their start (for baseline soil characteristics), while the content of As, Cd, Cr, Ni, and Pb was investigated in the SW-2016 experiment on two dates, that is: (1) before the start of the experiment; and (2) after wheat harvest. Sampling and sample preparation were as described in Section 2.4.2. Soil portions of about 300 g each were submitted to the chemical laboratory (accreditation number AB 696) for elemental analysis.

The C and N contents in soil were determined using the Vario Macro Cube Elementar (C,H,N) analyzer (Elementar Analysensysteme, Langenselbold, Germany). D-phenylalanine (C = 65.44%; N = 8.48%) was used as a standard solution. The contents of other elements were determined using an inductively coupled plasma-optical emission spectrometer (ICP–OES with a pneumatic nebulizer with an axial view—iCAP Duo Thermo Scientific, USA). An appropriate mass (0.5 g) of soil samples was digested in Teflon vessels (microwave oven Milestone MLS-1200, Sorisole, Bergamo, Italy) with 10 mL of aqua regia. After mineralization, all samples were diluted to 50 mL and then subjected to multi-elemental analyses using ICP-OES. Elemental determination was accomplished following all principles of measurement traceability. Certificated reference materials were also used to check the quality and metrological traceability. The detection levels (DL) of P, K, Mg, As, Cd, Cr, Ni and Pb in the soil material were 3.59, 2.55, 1.17, 0.5, 0.01, 0.035, 0.25, and 0.15 mg kg^–1^, respectively [78].

#### 2.4.4. Available Phosphorus (P) Content in Soil

The content of available P in soil was assayed in three experiments after the test crops were harvested. Sampling and sample preparation were as described in Section 2.4.2. The amount of available P present in the soil was determined in liquid soil extracts. The extract was carried out as follows: 1 g of soil and 50 mL of the extraction solution diluted two times were weighed into an Erlenmeyer flask and then shaken for 1.5 h on a 40 rpm shaker. According to the method described elsewhere [78], the obtained filtrate was subjected to mineralization and determination on ICP-OES. The extraction solution was prepared as follows: 120 g of calcium lactate was weighed into a 1 L volumetric flask, 750 mL of water and 40 mL of hydrochloric acid were added and heated, and then after the dissolution of the salt, one/two drops of chloroform were added, and the flask was then filled with the water till the mark.

#### 2.4.5. Abundance of Heterotrophic Bacteria and Fungi

Soil samples for analyses were taken from three experiments on two dates: (1) at wheat tillering and (2) at wheat heading. Sampling proceeded as described in Section 2.4.2. Each time, before switching to the next plot, soil samplers were sterilized with 96% ethanol (Chempur, Piekary Śląskie, Poland). Directly after collecting, each soil sample was thoroughly mixed with sterility maintained. Afterwards, small batches of soil material from each sample were placed in sterile 120 mL plastic pots, which were immediately transported to the microbiological laboratory. Heterotrophic bacteria were cultured on Tryptic Soy Agar (TSA, Merck KGaA, Darmstadt, Germany) and fungi on a Rose-Bengal Chloramphenicol (RBC, Merck KGaA, Darmstadt, Germany) agar (Table S3). The media were autoclave-sterilized (ASVE, Spółdzielnia Mechaników SMS, Warsaw, Poland) at 121 °C for 20 min, and were then cooled to 45–50 °C, thoroughly mixed and poured in the amount of 10 mL onto Petri plates with soil solution previously deposited on them (1 mL of 10–3, 10–4 and 10–5 dilutions). Each dilution was prepared in duplicate. Heterotrophic bacteria were incubated at 30 °C for 72 h, and fungi were incubated at 28 °C for five days. The emergent colonies of heterotrophic bacteria and fungi were counted (colony-forming units, CFU), calculated per 1 g of soil and log-transformed (Log10(CFU + 1)).

### 2.5. Calculations and Statistical Analysis

Based on PTE content in biobased fertilizers (Table 1; Cd content below DL was replaced by 0.01 mg kg^–1^), the PTE input per 1 ha with these agrochemicals and the potential increase in soil PTE content (per 1 kg of soil) were calculated. The weight of dry soil from the 0–30 cm-deep layer and 1 ha area was assumed to be 4500 t.

The data on wheat productivity and soil properties were processed using variance analysis (ANOVA) when its assumptions were met or the alternative Kruskal–Wallis test when ANOVA assumptions were not met. Verification of the distribution normality of variables and variance homogeneity was performed using the Shapiro–Wilk W test and Levene’s test, respectively. Duncan’s test or a multiple comparisons test were then applied to assess differences between objects. A median and a maximum value (Max) were determined for soil PTE content. In all statistical calculations for PTE, their contents below DL were replaced by values equal to the DL. The relationships between grain yield and yield structure elements and between grain yield and available P content in soil were expressed by simple correlation coefficients. The analyses were performed using Statistica 13.3 software [79].

## 3. Results and Discussion

### 3.1. Grain yield and Yield Structure Elements

The ultimate goal of fertilization is to increase yields [35]. In the current study, soil P reserves in experimental fields without P fertilization (P_0_) allowed wheat to produce yields of 6.02, 4.36 and 6.51 t ha^–1^ in experiments SW-2016, SW-2017 and WW-2017, respectively (Table 4). In each experiment, regardless of the fertilizer used, P application only at a dose of 17.6 kg ha^–1^ (SP_1_, AF_1_, and BF_1_) resulted in a significant yield increase compared to the relevant control treatment. The validity of increasing the P dose to 26.4 kg ha^–1^ was statistically confirmed only for SP application (SP_2_ versus SP_1_) in the WW-2017 experiment. However, in many cases, although a tendency to increase yield with increasing P dose was perceptible, the application of AF_3_ in the SW-2016 experiment and SP_3_ in experiments SW-2017 and WW-2017 no longer had only a statistical but also a biological justification. The weak response of wheat to the increase in P doses noted in the current study may have been a consequence of the P richness of the soil environment involved in the trials. Ram et al. [80] did not notice a significant response of wheat yield to the increase in a P dose from 13 to 26 kg ha^–1^ in an experiment set up on soil moderately rich in P, whereas a higher yielding capacity occurred on P deficient soils.

The yield-forming effect of BF was comparable to that of SP applied at the same P dose, while the AF_2_ treatment provided significantly lower wheat yield than SP_2_ in both the SW-2017 and WW-2017 experiments. Under field conditions, the more beneficial yield-forming effect of BF in relation to AF was mostly manifested by trends (weak or more pronounced) and was only confirmed for BF_2_ against AF_2_ in the WW-2017 experiment. It was also observed that the beneficial effect of BF compared to AF was more evident under poorer habitat conditions (worse previous crop in SW-2017 and WW-2017, lower soil P content in WW-2017).

Reports on the satisfying yield stimulating effect of fertilizers produced from SSA have been published previously [64,68,81]. However, under-performance was also noted [82,83]. The meta-analysis performed by Huygens and Saveyn [36] suggests that P-fertilizers derived from SSA may compare in agronomic efficiency with mined and synthetic P-fertilizers, but it is dependent on the raw material applied, possible post-combustion manufacturing processes and the length of the plant growing season. Despite their low water solubility, recycled P-fertilizers are considered to be a valuable alternative for traditional ones in conventional and organic European agriculture [35,36].

Studies to date on the role of PSM in improving the yield-forming efficiency of fertilizer P sources do not yet provide clear answers. In relation to conventional P sources and fertilizers, both positive effects [60,80,84,85] and lack of the expected interaction were noted [84,86]. Although it was proven in laboratory research that PSM are efficient in solubilizing P from SSA [62], many studies, both pot [84,86,87] and field [88,89], demonstrate the poor contribution of bioeffectors (microbes) in increasing the yield-forming efficiency of SSA-based P-fertilizers. The findings from the current study are promising, especially from the WW-2017 experiment, although they are also in line with the opinions that more studies are required to understand agronomic effects of SSA [90] and to fully recognize the necessity, opportunities and conditions for increasing these effects through PSM activity [31,88,89]. Since many technologies are at the pilot or laboratory scale research stage [37], comprehensive field studies are in particular demand [31,45].

In the current study, the tested fertilizer treatments did not differentiate wheat spike density and grain number per spike in all the experiments, while P fertilization usually increased 1000 grain weight (TGW) relative to P_0_ (except for SP_1_, AF_1_, and AF_2_ in experiment WW-2017—only an increasing tendency was observed). The applied fertilizers and P doses influenced TGW in the same way. In all three experiments, among the elements of yield structure, TGW was the strongest contributor to yield formation (Table 5). In experiment SW-2016, the yield was also positively correlated with spike density, with no relationship with grain number per spike. Spike density and grain number per spike did not correlate with yield in the SW-2017 and WW-2017 experiments. The literature is not clear on the contribution of yield structure components in forming the yield of individual cereal species [91]. It is argued that unambiguous determination of the effect of yield structure components on yield is difficult because it is determined by both genetic factors (species, cultivar) and environmental conditions [92].

### 3.2. Soil pH

Before the start of the SW-2016 and SW-2017 experiments (Table 3), soil pH in the respective fields fell within the range corresponding to the highest P bioavailability (5.5–7.0) [93]. In experiment WW-2017, the initial soil pH was below 5.5, indicating that P availability was limited by P incorporation into aluminum (Al) or iron (Fe) compounds [94,95].

No differences in pH induced by applied P fertilization were found in any experiment either at the tillering stage of wheat or after wheat harvest (Table 6). In contrast, pot experiments conducted by Rosyadi [96] and Zalewska et al. [97] showed an increase in soil pH after test plant harvest as a result of SSA application compared to no P treatments and conventional mineral P fertilizers (triple superphosphate (TSP) and monocalcium phosphate, respectively). Rosyadi [96] explains these differences by way of the higher Ca load in SSA and the nature of the ashing process, where CO_2_ is removed and alkaline CaO is generated, as well as by the potential release of protons during TSP hydrolysis. This deacidifying/alkalizing effect of SSA [98] was not pronounced in the present studies conducted under field conditions, i.e., in an open, only partially controlled system. Similar results were obtained by Jastrzębska et al. in field studies with other SSA-based fertilizers [99,100].

It is also worth noting that, in the present study, no change in soil pH was observed under the influence of *B. megaterium* bacteria introduced with BF. Based on *B. megaterium* biology [101] and previous laboratory studies [62] indicating that the growth of *B*. *megaterium* cultured on SSA was accompanied by a lowering of medium pH, the possibility of a decrease in soil pH resulting from the activity of *B.megaterium* introduced with BF was assumed. However, it seems that the abundance of *B. megaterium* introduced with BF was too low to affect the soil pH value under field conditions in confrontation with the soil buffering properties [102], or with the possible bacterial response to the encountered adverse environmental conditions (fast sporulation [103]). In other studies by Jastrzębska et al. conducted in field conditions, SSA-based fertilizers activated by *B.megaterium* did not increase the abundance of *B.megaterium* in the soil [104] and did not change the soil pH [99]. Similarly, Zhao et al. 2021 [85] reported that, compared to conventional fertilization (chemical fertilizer + manure), the addition of *B.megaterium* did not change soil pH after cucumber harvest.

In experiment SW-2016, soil pH was statistically stable during the growing season, whereas differential seasonal changes in soil pH, independent of fertilizer type and P rate, were found in experiments SW-2017 and WW-2017. Although soils have their specific buffering capacities [102], seasonal fluctuations in soil pH of 0.2–0.3 of a pH unit are, in fact, not unusual [105]. Many factors are cited among their causes, including fertilizer type, rate and time of application, organic matter, plant root and bacterial activity, soil moisture [105], plant uptake of nutrients in the form of cations and anions [106], and nutrient leaching or volatilization (depending on the element and the form of its compounds) [107,108]. A more detailed discussion of seasonal changes in soil pH is presented in the Supporting Information.

### 3.3. Content of Potentially Toxic Elements in the Soil

The contents of major potentially toxic elements (PTE), i.e., As, Cd, Cr, Ni and Pb, that were determined in the soil in the SW-2016 experiment (Table 7), fell within the ranges considered natural for Polish and regional conditions and did not exceed the permitted levels for arable lands in Poland (see Table S4). Even the maximum values found in the present study were within the geochemical background, except for Cd, of which the maximum content was slightly higher (1.149 vs. 1.000 mg kg^–1^). The median values for all studied PTE were lower than the averages.

Since PTE were present in the elemental composition of the biobased fertilizers (Table 1), the possibility of an increase in soil PTE content as a result of AF and BF application (especially compared to the P_0_ treatment) was considered. The amounts of PTE that entered the soil with AF and BF fertilizers were so small (Table 8) that the enrichment of soil PTE ended up being negligible for statistical analysis. No changes in soil PTE content compared to the initial state were proven, nor were differences due to the influence of the fertilizer factor. Regardless of the P dose, AF and BF, just like SP, did not significantly change the content of these elements compared to the control (P_0_).

Previous reports indicate a possible increase in soil PTE content as a result of the long-term application of P fertilizers, both conventional and from renewable resources, if they carried high PTE amounts [110,111]. Not only the amount of PTE carried with the fertilizer, but also which PTE the fertilizer contains seems to be important. For example, it is believed that under normal cropping practices, As accumulation in soils is insignificant [110,112], while the application of Cd-containing P-fertilizers could, over time, cause Cd to accumulate in soil and therefore increases the accompanying risks [110]. On the other hand, according to Uprety et al. [113], common cropping practices with the application of organic and inorganic fertilizers do not substantially enrich soils in As, Cd, Cr, Ni, and Pb, even after they have been applied for more than 50 years.

Chen et al. [110] found an increase in soil Pb content after a 10-year application of 2.35–47.0 g Pb along with 25–400 kg ha^–1^ P (statistically the same increase regardless of the P dose), but no increase in As and Cd content when applied at rates up to 42.2 g and 1.01 g (with 200 kg ha^–1^ P) and in Cr and Ni content when applied at a rate up to 42.4 g and 21.7 g (with 400 kg ha^–1^ P), respectively. A point worth noting is that, except for Pb, the amounts of PTE introduced into the soil together with AF and BF fertilizers in the present study (Table 8) were substantially lower. Another study by Jastrzębska et al. [114] based on several one-year experiments with different SSA-containing fertilizers also brought promising findings; however, considering the nature of PTE, further long-term studies are called for.

### 3.4. Available Phosphorus (P) Content in Soil

According to the standard Polish classification of plant-available P by Egner-Riehm [115], the content of available P in soil (Table 9) should be classified as low to medium in experiment SW-2016, as a medium in experiment SW-2017, and as low in experiment WW-2017. The latter was due not only to the relatively low total P content in the soil but also to the soil pH limiting P bioavailability [95].

In all three experiments, P fertilization increased or tended to increase the available P content in the soil. Only a trend toward enhanced available P was observed in experiments SW-2016 and WW-2017 after the application of SP_1_, AF_1_, AF_2_, AF_3_, and BF_1_ treatments. Higher rates of SP (SP_2_, SP_3_) and BF (BF_2_, BF_3_) in these experiments, as well as all P fertilization treatments in experiment SW-2017 resulted in a significant increase in available P in the soil. In all experiments, the highest available P content in soil was recorded after SP_3_ application. This is mostly the result of applying a relatively high dose of P in a readily available form [116] that was not fully utilized by the plants (Table 4).

When comparing the same P amounts (doses) introduced with different fertilizers, lower amounts of available P were found after the application of AF_3_ and BF_3_ in SW-2017 and after AF_3_ in WW-2017, compared to SP_3_, respectively in the relevant experiments. The differences for SP_3_ versus AF_3_ are explained by the lower solubility of P compounds in the waste raw material [44], while the differences for SP_3_ versus BF_3_ are rather due to the unused part of available P by plants after SP_3_ application (see Table 4). In other cases, the available P content under the influence of AF and BF did not differ from that formed by SP applied at the same P dose.

The performance of BF against AF did not prove spectacular, but it should be rated as moderately optimistic. In the SW-2016 experiment, the solubilizing activity of *B. megaterium* contained in BF was shown only by a tendency to increase available P content compared to the effect of AF applied at the same P dose. In the SW-2017 experiment, a positive tendency under BF_1_ versus AF_1_ was also noted, but a significant increase in available P content was observed under BF_2_ and BF_3_ in comparison to AF_2_ and AF_3_, respectively. In contrast, in the WW-2017 experiment, BF_1_ and BF_2_ treatments resulted in a trend of higher available P content relative to AF_1_ and AF_2_, while higher available P content was confirmed under BF_3_ relative to AF_3_. These effects are also related to the fact that some portion of the P dissolved by *B. megaterium* was not used by the plants to build a higher yield. However, in all experiments, a positive correlation was found between the content of available P in the soil after wheat harvest and wheat grain yield (Table 10).

The high solubilization activity of *B. megaterium* has been proven in previous studies [117], as well as its contribution to increasing available P in soil [118]. This bacterial strain is mentioned among commercially used species of PSM [119]. In laboratory tests, Wyciszkiewicz et al. [49] demonstrated the potential of using *B. megaterium* to solubilize P compounds of SSA, although the solubilization efficiency was lower than with animal bones as raw material. Since the rate and the intensity of the P solubilization process are dependent on many factors (e.g., pH, temperature, moisture and others) [119], this activity of *B. megaterium* can be strongly modified under field conditions where many environmental parameters are uncontrolled, and over the period of fertilization which is often unfavorable for microbes. The results of the current study are consistent with the claim that beneficial soil bacteria under nutrient-poor conditions are more likely to act as nutrient solubilizers (see WW-2017 in Table 9) but do not prove their performance as plant growth enhancers under nutrient-rich soil conditions (see Table 4 and Table 9) [120]. For comparison, Zhao et al. [85] reported that, relative to conventional fertilization (chemical fertilizer + manure), the addition of *B. megaterium* significantly increased cucumber yield and the dry weight of cucumber fruit and roots without increasing the soil available P content. It can also be assumed that the abundance of bacteria introduced into the soil with BF in the current study was too low, or they were not competitive enough to proliferate efficiently and intensify P release. Raymond et al. [89] suggested that inoculation with the P-solubilizing fungus *Penicillium bilaiae* did not prove to be a suitable strategy to enhance P availability from SSA, probably due to the limited competitiveness of the fungus in the soil.

Further research on PSM strain selection and their ratio in waste-based fertilizer formulations, as well as a better understanding of PSM interactions with feedstock P under natural (field) conditions would be supportive in developing this alternative P-fertilizing approach.

### 3.5. Abundance of Heterotrophic Bacteria and Fungi

The numbers of heterotrophic bacteria and fungi found in the soil of the conducted experiments (Table 11) were within the ranges corresponding to the microbiological characteristics of Polish arable soils [121]. The tested P fertilization treatments did not differentiate the abundance of these microorganisms in any of the experiments and on none of the analysis dates adopted.

Studies by other authors have shown that the introduction of P into the soil in the form of mineral fertilizers, as well as the type of fertilizer, did not change the abundance of soil microorganisms relative to the control (P_0_) [122,123,124,125]; however, both mineral P application and fertilizer type (e.g., phosphate rock vs. triple superphosphate) altered the structure and diversity of the microbial community [124,125,126]. Moreover, for the latter, contradictory effects were observed [125].

Although the relationships between native soil microorganisms and introduced microorganisms (their compatibility or incompatibility) are not fully recognized [127], some studies have shown that both the abundance and structure of indigenous communities can be altered by allochthonous organisms [128] through competition, activity stimulation and other microbe–microbe interactions [61]. In the present study, no change in the abundance of heterotrophic bacteria and soil fungi under the influence of *B. megaterium* introduced into the soil with BF was observed in any of the field experiments. This may be due to the relatively low abundance of *B. megaterium* in the BF formulation. Moreover, the activation of the lyophilized cells and their proliferation strongly depended on the environmental conditions, mainly the proper soil moisture and temperature. In other studies by Jastrzębska et al. [99,100], similar results were obtained when *B. megaterium* was a component of suspension or granular fertilizers from SSA and/or animal bones. In contrast, Ali et al. [129] reported that *Bacillus subtilis* applied as a biofertilizer alone or in compilation with triple superphosphate increased the number of bacteria in soil under wheat. Zhao et al. [85] found that the application of *B. megaterium* improved the richness of bacterial and fungal communities, increased the relative abundances of the beneficial bacterial genera and fungal orders, and suppressed the development of pathogenic bacterial genus in soil under cucumber grown in the plastic shed system. It should be added that in the studies by Ali et al. [129] and Zhao et al. [85], active bacteria were applied in large quantities. Analysis of the structure and biodiversity of soil microbial communities under the influence of fertilizers and biofertilizers from waste biomass is a further research challenge for the authors of the present study.

The differences in bacterial abundance between analysis dates found in the present study resulted from the rainfall quantity occurring in the period prior to analysis in each experiment and from the corresponding soil moisture. Seasonal variation in fungal abundance was not observed. Fungi are considered more tolerant of soil drought than bacteria. It is accepted that bacteria show no activity at less than 30% environmental moisture, while fungal growth ceases at less than 15% moisture [75].

## 4. Conclusions

Under field conditions, the effectiveness of BF fertilizer was comparable with SP fertilizer regarding the yield-forming potential. In comparison with SP and BF fertilizers, the AF fertilizer (without bacteria) usually had a slightly weaker yield-forming ability. AF and BF fertilizers resulted in the same or a lower level of soil available P as SP applied at the same P rate. The slightly higher effectiveness of BF relative to AF at increasing yield and soil available P was evident under poorer habitat conditions. Both AF and BF, applied at the P rate up to 35.2 kg ha^–1^ (similar to SP), did not affect the pH level of soil under the test plant, did not increase As, Cd, Cr, Ni, and Pb in the soil, and did not alter the abundance of heterotrophic bacteria or fungi in the soil. These findings demonstrate that such recycled agrochemicals can be an alternative to conventional P-fertilizers under phosphate rock shortage or at least partially replace them. The tested fertilizers are admittedly less P-rich than commercial fertilizers but additionally contain numerous macro- and micronutrients. The comparable results obtained for AF and BF put into question the need for biological activation of the waste biomass-based preparation. However, the advantageous trends outlined should inspire further work towards increasing the proportion of *B. megaterium* in BF or searching for more effective biological activators. This promising alternative P-fertilizing approach also needs to be verified in long-term field studies, especially with respect to the potential accumulation of PTE introduced into the soil with waste-based fertilizers.

## Figures and Tables

**Table 1:** Elemental composition of the biobased fertilizers under study ^1^.

Element	Unit	AF		BF	
		2016	2017	2016	2017
P	% mass.	8.68	5.40	9.55	4.95
N		2.89	3.44	2.87	3.15
K		1.09	0.62	1.16	0.67
Ca		13.4	14.2	14.6	12.3
Mg		1.54	0.79	1.70	0.78
S		0.56	0.47	0.56	0.40
C		12.5	16.5	13.9	18.1
Fe	g kg^–1^	26.9	11.4	29.0	11.3
Al		23.7	11.3	25.5	12.1
Zn		3.14	1.09	3.29	0.99
As	mg kg^–1^	31.4	15.5	20.0	20.5
Cd		<0.01	0.660	0.345	0.742
Cr		54.7	63.9	62.9	59.1
Cu		778	334	850	334
Ni		54.8	28.5	62.6	21.2
Pb		19.9	0.920	21.8	4.53
B		71.3	41.1	74.1	57.6
Ba		349	162	382	168
Co		14.0	5.24	16.2	4.24
Mn		562	299	609	437
Mo		35.3	9.25	23.7	13.9

^1^ According to the Department of Advanced Material Technologies of the Wrocław University of Science and Technology, Wrocław, Poland [71].

**Table 2:** Fertilization treatments tested and their symbols.

P-Fertilizers	P Doses, kg ha^–1^			
	0	17.6	26.4	35.2
No phosphorus fertilizer	P_0_			
Superphosphate (SP)		SP_1_	SP_2_	SP_3_
Biomass-based fertilizer (AF)		AF_1_	AF_2_	AF_3_
Biomass-based biofertilizer (BF)		BF_1_	BF_2_	BF_3_

**Table 3:** Soil characteristics before the start of the experiments.

Experiment	Soil Type	Soil Texture	pH in KCl	Total, g kg^–1^				
				C	N	P	K	Mg
SW-2016	Luvisols ^1^	sandy clay loam	6.28	8.53	1.42	0.61	2.98	2.02
SW-2017	Luvisols	sandy clay loam	6.23	8.48	1.34	0.60	3.14	1.94
WW-2017	Luvisols	sandy loam	4.98	6.48	1.01	0.49	2.95	1.88

^1^ Classification according to World reference base for soil resources 2014 [72].

**Table 4:** Wheat grain yield and its components.

Item	Treatments	SW-2016	SW-2017	WW-2017
Grain yield, t ha^–1^	P_0_	6.02 c ^1^	4.36 c	6.51 c
	SP_1_	6.40 ab	5.45 ab	7.42 b
	SP_2_	6.53 ab	5.71 a	8.08 a
	SP_3_	6.70 a	5.38 ab	7.72 ab
	AF_1_	6.32 b	5.10 b	7.36 b
	AF_2_	6.40 ab	5.13 b	7.47 b
	AF_3_	6.39 ab	5.23 ab	7.66 ab
	BF_1_	6.38 ab	5.28 ab	7.75 ab
	BF_2_	6.48 ab	5.45 ab	8.00 a
	BF_3_	6.59 ab	5.63 ab	8.03 a
Spike density,	P_0_	482	450	457
No m^–2^	SP_1_	489	545	481
	SP_2_	497	549	509
	SP_3_	499	513	506
	AF_1_	489	477	487
	AF_2_	493	512	480
	AF_3_	491	451	482
	BF_1_	491	449	480
	BF_2_	497	523	489
	BF_3_	501	494	478
Grain number per spike, No	P_0_	28	22	31
	SP_1_	31	27	33
	SP_2_	34	27	38
	SP_3_	31	30	33
	AF_1_	33	25	31
	AF_2_	33	24	35
	AF_3_	31	27	32
	BF_1_	31	29	32
	BF_2_	31	26	38
	BF_3_	32	26	36
1000 grain weight, g	P_0_	42.8 b	41.2 b	45.5 b
	SP_1_	44.8 a	43.1 a	47.2 ab
	SP_2_	44.9 a	43.9 a	48.8 a
	SP_3_	45.3 a	43.5 a	48.5 a
	AF_1_	44.8 a	42.8 a	47.5 ab
	AF_2_	44.8 a	43.2 a	47.7 ab
	AF_3_	44.9 a	43.9 a	48.1 a
	BF_1_	44.8 a	43.1 a	48.2 a
	BF_2_	45.1 a	43.5 a	48.3 a
	BF_3_	45.3 a	44.0 a	48.6 a

^1^ different letters within columns indicate significant differences at *p* ≤ 0.05, no letters—no significant differences.

**Table 5:** Relationship of wheat yield to yield structure elements—simple correlation coefficients.

Yield Structure Element	SW-2016 (*n* = 40)	SW-2017 (*n* = 40)	WW-2017 (*n* = 40)
Spike density	0.347 *	0.199	0.251
Grain number per spike	0.068	0.276	0.194
1000 grain weight	0.657 **	0.470 *	0.570 **

* value significant at *p* ≤ 0.05; ** value significant at *p* ≤ 0.01.

**Table 6:** Soil pH in the experiments, in 1 M KCl ^1^.

Treatments	SW-2016		SW-2017		WW-2017	
	at Tillering	after Harvest	at Tillering	after Harvest	at Tillering	after Harvest
P_0_	6.24	6.17	6.08	6.20	5.15	5.13
SP_1_	6.11	6.25	5.95	6.25	5.25	5.23
SP_2_	6.21	6.20	5.95	6.23	5.13	5.15
SP_3_	6.19	6.05	6.00	6.20	5.30	5.18
AF_1_	6.17	6.37	5.93	6.28	5.35	5.33
AF_2_	6.27	6.25	6.05	6.45	5.20	5.15
AF_3_	6.26	6.36	6.25	6.30	5.33	5.25
BF_1_	6.14	6.18	6.05	6.30	5.30	5.28
BF_2_	6.13	6.28	6.05	6.33	5.10	4.98
BF_3_	6.28	6.22	6.15	6.38	5.08	5.10
Average	6.20	6.23	6.05↓ ^2^	6.29↑	5.22↑	5.18

^1^ no significant differences between treatments; ^2^ arrows indicate significant increase or decrease in relation to the state at previous analysis date.

**Table 7:** Soil content of potentially toxic elements (PTE) after spring wheat (2016) harvest, mg kg^–1^ of soil DM ^1^.

Treatments	As	Cd	Cr	Ni	Pb
P_0_	1.70	0.330	19.9	12.33	2.04
SP_1_	0.98	0.164	20.2	9.00	1.85
SP_2_	0.71	0.188	21.0	10.17	0.60
SP_3_	1.30	0.234	20.6	6.91	0.15
AF_1_	1.19	0.318	22.3	5.35	1.43
AF_2_	2.33	0.024	22.8	11.02	0.60
AF_3_	2.06	0.126	22.3	10.67	2.97
BF_1_	0.89	0.082	21.7	5.29	1.82
BF_2_	1.22	0.010	19.6	7.34	0.46
BF_3_	1.48	0.210	21.4	7.83	0.50
Average	1.39	0.169	21.2	8.59	1.24
Median	0.67	0.046	20.9	7.70	0.15
Max	5.63	1.149	31.8	16.59	11.4

^1^ no significant differences between treatments and no significant changes relative to the baseline state.

**Table 8:** Quantities of PTE introduced into soil with biomass-based fertilizers—range between the values for the lowest (17.6 kg ha^–1^) and the highest (35.2 kg ha^–1^) P dose.

Fertilizers	As	Cd	Cr	Ni	Pb
	Input per 1 ha, g				
AF	6.37–12.73	0.002–0.004 ^1^	11.09–22.18	11.11–22.22	4.04–8.07
BF	3.69–7.37	0.064–0.127	11.59–23.18	11.54–23.07	4.02–8.04
Limit values per year ^2^	n.s.	150	n.s.	3000	15,000
	**Potential increase in soil content, µg kg^–1^ of soil DM**				
AF	1.41–2.83	0.0005–0.0009 ^1^	2.46–4.93	2.47–4.94	0.90–1.79
BF	0.82–1.64	0.0141–0.0283	2.58–5.15	2.56–5.13	0.89–1.79

^1^ potentially maximum values; ^2^ according to Final Implementation Report for Directive 86/278/EEC on Sewage Sludge: 2013–2015 [109]; n.s.—not set.

**Table 9:** Available P content in soil after wheat harvest, mg kg^–1^.

Treatments	SW-2016	SW-2017	WW-2017
P_0_	42.1 c ^1^	48.69 e	25.2 d
SP_1_	44.1 abc	51.22 d	27.0 bcd
SP_2_	46.0 ab	53.12 bc	29.7 ab
SP_3_	47.7 a	55.70 a	32.4 a
AF_1_	43.6 bc	50.56 d	26.1 cd
AF_2_	43.4 bc	51.48 cd	26.4 bcd
AF_3_	44.8 abc	51.62 cd	26.1 cd
BF_1_	44.5 abc	51.28 cd	28.0 bcd
BF_2_	46.1 ab	53.81 b	28.8 bc
BF_3_	46.9 ab	53.98 b	29.9 ab

^1^ different letters within columns indicate significant differences at *p* ≤ 0.05.

**Table 10:** Relationship between available P content in soil and wheat grain yield–simple correlation coefficients.

SW-2016 (*n* = 40)	SW-2017 (*n* = 40)	WW-2017 (*n* = 40)
0.466 *	0.504 **	0.423 *

* value significant at *p* ≤ 0.05; ** value significant at *p* ≤ 0.01.

**Table 11:** Abundance of heterotrophic bacteria and fungi in the 0–30 cm layer of soil, Log_10_(CFU + 1) per 1 g of soil DM ^1^.

Treatments	SW-2016		SW-2017		WW-2017	
	Wheat Development Stages					
	Tillering	Heading	Tillering	Heading	Tillering	Heading
Bacteria						
P_0_	6.97	6.19	6.03	7.03	6.64	6.16
SP_1_	6.87	6.25	6.22	6.89	6.67	6.10
SP_2_	6.78	6.19	6.05	6.85	6.65	6.16
SP_3_	6.81	6.14	6.07	7.13	6.69	6.20
AF_1_	6.89	6.29	5.96	7.12	6.80	6.33
AF_2_	6.77	6.12	5.89	7.25	6.63	6.22
AF_3_	6.77	6.14	5.98	6.69	6.77	6.20
BF_1_	6.89	6.26	6.06	7.22	6.70	6.26
BF_2_	6.82	6.15	6.11	7.20	6.71	6.09
BF_3_	6.86	6.23	6.10	7.07	6.76	6.21
Average	6.85	6.20↓ ^2^	6.05	7.08↑	6.71	6.20↓
**Fungi**						
P_0_	4.46	4.34	4.30	4.41	4.56	4.54
SP_1_	4.41	4.53	4.36	4.57	4.64	4.70
SP_2_	4.40	4.45	4.38	4.53	4.62	4.67
SP_3_	4.48	4.58	4.34	4.49	4.54	4.62
AF_1_	4.48	4.53	4.46	4.69	4.61	4.66
AF_2_	4.43	4.57	4.48	4.64	4.56	4.59
AF_3_	4.57	4.66	4.38	4.56	4.52	4.62
BF_1_	4.58	4.57	4.52	4.68	4.57	4.59
BF_2_	4.40	4.53	4.41	4.52	4.65	4.54
BF_3_	4.45	4.51	4.41	4.51	4.54	4.67
Average	4.46	4.53	4.41	4.57	4.59	4.63

^1^ no significant differences between treatments; ^2^ arrows indicate significant increase or decrease in relation to the state at previous analysis date.

## Data Availability

Not applicable.

## Supplementary Materials

The following supporting information can be downloaded at: https://www.mdpi.com/article/10.3390/molecules27092769/s1, Table S1: Basic agricultural information for the experiments; Table S2: Atmospheric precipitation and air temperature during the study period according to the Meteorological Station in Bałcyny, Poland; Table S3: Microbiological culture media composition; Discussion on seasonal changes in soil pH in the experiments; Table S4: Reference values for soil PTE content; References [94,106,130,131,132,133,134,135,136,137] are used in Supplementary Materials.

## Sample Availability

Samples of the compounds are not available from the authors.

## References

[1] Current World Population World Population Clock. http://www.worldometers.info/world-population/.

[2] FAO (2019). FAOSTAT Database. https://www.fao.org/faostat/en/#data.

[3] Calabi-Floody M., Medina J., Rumpel C., Condron L.M., Hernandez M., Dumont M., Mora M.d.l.L., Sparks D.L. (2018). Smart Fertilizers as a Strategy for Sustainable Agriculture. Advances in Agronomy.

[4] OECD, FAO (2020). OECD-FAO Agricultural Outlook 2020–2029.

[5] Sofyane A., Ablouh E., Lahcini M., Elmeziane A., Khouloud M., Kaddami H., Raihane M. (2021). Slow-release fertilizers based on starch acetate/glycerol/polyvinyl alcohol biocomposites for sustained nutrient release. Mater. Today Proc..

[6] Schröder P., Sauvêtre A., Gnädinger F., Pesaresi P., Chmeliková L., Doğan N., Gerl G., Gökçe A., Hamel C., Millan R. (2019). Discussion paper: Sustainable increase of crop production through improved technical strategies, breeding and adapted management—A European perspective. Sci. Total Environ..

[7] Ishangulyyev R., Kim S., Lee S.H. (2019). Understanding Food Loss and Waste—Why Are We Losing and Wasting Food?. Foods.

[8] Aziz M.Z., Naveed M., Abbas T., Siddique S., Yaseen M., Farooq M., Pisante M. (2019). Alternative Fertilizers and Sustainable Agriculture. Innovations in Sustainable Agriculture.

[9] Tong Z., Quan G., Wan L., He F., Li X. (2019). The Effect of Fertilizers on Biomass and Biodiversity on a Semi-Arid Grassland of Northern China. Sustainability.

[10] Bindraban P.S., Dimkpa C., Nagarajan L., Roy A., Rabbinge R. (2015). Revisiting fertilisers and fertilisation strategies for improved nutrient uptake by plants. Biol. Fertil. Soils.

[11] Ogeto R.M., Jiong G. (2019). Fertilizer underuse in Sub Saharan Africa: Evidence from maize. J. Agric. Econ. Dev.

[12] Ren C., Jin S., Wu Y., Zhang B., Kanter D., Wu B., Xi X., Zhang X., Chen D., Xu J. (2021). Fertilizer overuse in Chinese smallholders due to lack of fixed inputs. J. Environ. Manag..

[13] Chandini K.R., Kumar R., Prakash O. (2019). The impact of chemical fertilizers on our environment and ecosystem. Research Trends in Environmental Sciences.

[14] Wu H., Ge Y. (2019). Excessive application of fertilizer, agricultural non-point source pollution, and farmers’ policy choice. Sustainability.

[15] Bhushan L.S., Pathma J. (2021). Impact of agro-chemicals on environment: A global perspective. Plant Cell Biotechnol. Mol. Biol..

[16] Sabry A.K. (2015). Synthetic fertilizers; role and hazards. Fertil. Technol..

[17] Ahmed M., Rauf M., Mukhtar Z., Saeed N.A. (2017). Excessive use of nitrogenous fertilizers: An unawareness causing serious threats to environment and human health. Environ. Sci. Pollut. Res..

[18] Sharma N., Singhvi R. (2017). Effects of chemical fertilizers and pesticides on human health and environment: A review. Int. J. Agric. Environ. Biotechnol..

[19] Jastrzębska M., Kostrzewska M., Saeid A., Chojnacka K., Saeid A. (2022). Conventional agrochemicals: Pros and cons. Smart Agrochemicals for Sustainable Agriculture.

[20] Svanbäck A., McCrackin M.L., Swaney D.P., Linefur H., Gustafsson B.G., Howarth R.W., Humborg C. (2019). Reducing agricultural nutrient surpluses in a large catchment—Links to livestock density. Sci. Total Environ..

[21] Sigurnjak I., Michels E., Crappé S., Buysens S., Tack F.M.G., Meers E. (2016). Utilization of derivatives from nutrient recovery processes as alternatives for fossil-based mineral fertilizers in commercial greenhouse production of *Lactuca sativa* L.. Sci. Hortic..

[22] Velenturf A.P.M., Purnell P. (2021). Principles for a sustainable circular economy. Sustain. Prod. Consum..

[23] Kirchherr J., Reike D., Hekkert M. (2017). Conceptualizing the circular economy: An analysis of 114 definitions. Resour. Conserv. Recycl..

[24] Zink T., Geyer R. (2017). Circular Economy Rebound. J. Ind. Ecol..

[25] Velasco-Muñoz J.F., Mendoza J.M.F., Aznar-Sánchez J.A., Gallego-Schmid A. (2021). Circular economy implementation in the agricultural sector: Definition, strategies and indicators. Resour. Conserv. Recycl..

[26] Chojnacka K., Moustakas K., Witek-Krowiak A. (2020). Bio-based fertilizers: A practical approach towards circular economy. Bioresour. Technol..

[27] Chojnacka K., Gorazda K., Witek-Krowiak A., Moustakas K. (2019). Recovery of fertilizer nutrients from materials—Contradictions, mistakes and future trends. Renew. Sustain. Energy Rev..

[28] Buckwell A., Nadeu E. (2016). Nutrient Recovery and Reuse (NRR) in European Agriculture: A Review of the Issues, Opportunities, and Actions.

[29] Lupton S. (2017). Markets for waste and waste–derived fertilizers. An empirical survey. J. Rural Stud..

[30] Jedelhauser M., Binder C.R. (2018). The spatial impact of socio-technical transitions–The case of phosphorus recycling as a pilot of the circular economy. J. Clean. Prod..

[31] Jastrzębska M., Kostrzewska M.K., Saeid A. (2019). Can Phosphorus from Recycled Fertilisers Replace Conventional Sources? An Agronomic Evaluation in Field-Scale Experiments on Temperate Luvisols. Appl. Sci..

[32] USGS (2022). Mineral commodity summaries 2022. Phosphate Rock.

[33] EC (2014). Report on critical raw materials for the EU. Report of the Ad-Hoc Working Group on Defining Critical Raw Materials.

[34] EC (2017). Study on the Review of the List of Critical Raw Materials.

[35] Kratz S., Vogel C., Adam C. (2019). Agronomic performance of P recycling fertilizers and methods to predict it: A review. Nutr. Cycl. Agroecosystems.

[36] Huygens D., Saveyn H.G.M. (2018). Agronomic efficiency of selected phosphorus fertilisers derived from secondary raw materials for European agriculture. A meta-analysis. Agron. Sustain. Dev..

[37] Günther S., Grunert M., Müller S. (2018). Overview of recent advances in phosphorus recovery for fertilizer production. Eng. Life Sci..

[38] EU (2019). Regulation (EU) 2019/1009 of the European Parliament and of the Council of 5 June 2019 laying down rules on the making available on the market of EU fertilising products and amending Regulations (EC) No 1069/2009 and (EC) No 1107/2009 and repealing Regulation (EC) No 2003/2003 (Text with EEA relevance). Off. J. Eur. Union.

[39] Izydorczyk G., Mikula K., Skrzypczak D., Trzaska K., Moustakas K., Witek-Krowiak A., Chojnacka K. (2021). Agricultural and non-agricultural directions of bio-based sewage sludge valorization by chemical conditioning. Environ. Sci. Pollut. Res..

[40] Fijalkowski K., Rorat A., Grobelak A., Kacprzak M.J. (2017). The presence of contaminations in sewage sludge—The current situation. J. Environ. Manag..

[41] Hudcová H., Vymazal J., Rozkošný M. (2019). Present restrictions of sewage sludge application in agriculture within the European Union. Soil Water Res..

[42] Gorazda K., Tarko B., Wzorek Z., Kominko H., Nowak A.K., Kulczycka J., Henclik A., Smol M. (2017). Fertilisers production from ashes after sewage sludge combustion—A strategy towards sustainable development. Environ. Res..

[43] ME-PL (2020). Regulation of the Minister of the Environment, Republic of Poland, Catalogue of waste. J. Laws.

[44] Herzel H., Krüger O., Hermann L., Adam C. (2016). Sewage sludge ash—A promising secondary phosphorus source for fertilizer production. Sci. Total Environ..

[45] Smol M., Adam C., Kugler S.A. (2020). Thermochemical treatment of Sewage Sludge Ash (SSA)-potential and perspective in Poland. Energies.

[46] Donatello S., Cheeseman C.R. (2013). Recycling and recovery routes for incinerated sewage sludge ash (ISSA): A review. Waste Manag..

[47] Smol M., Kulczycka J., Kowalski Z. (2016). Sewage sludge ash (SSA) from large and small incineration plants as a potential source of phosphorus—Polish case study. J. Environ. Manag..

[48] Van Kauwenbergh S.J. (2010). World Phosphate Rock Reserves and Resources.

[49] Wyciszkiewicz M., Sojka M., Saeid A. (2019). Production of phosphorus biofertilizer based on the renewable materials in large laboratory scale. Open Chem..

[50] Erwiha G.M., Ham J., Sukor A., Wickham A., Davis J.G. (2020). Organic Fertilizer Source and Application Method Impact Ammonia Volatilization. Commun. Soil Sci. Plant Anal..

[51] Choi J.-H., Lamshöft M., Zühlke S., Park J.-H., Rahman M.M., El-Aty A.M.A., Spiteller M., Shim J.-H. (2014). Determination of anxiolytic veterinary drugs from biological fertilizer blood meal using liquid chromatography high-resolution mass spectrometry. Biomed. Chromatogr..

[52] Kowalski Z., Makara A., Banach M. (2011). Krew zwierzęca, metody jej przetwarzania i zastosowanie. Technol. Trans. Chem..

[53] Rosen C.J., Allan D.L. (2007). Exploring the Benefits of Organic Nutrient Sources for Crop Production and Soil Quality. HortTechnol. Hortte.

[54] Saeid A., Wyciszkiewicz M., Jastrzebska M., Chojnacka K., Gorecki H. (2015). A concept of production of new generation of phosphorus-containing biofertilizers. BioFertP project. Przem. Chem..

[55] Khan M.S., Zaidi A., Wani P.A. (2007). Role of phosphate-solubilizing microorganisms in sustainable agriculture—A review. Agron. Sustain. Dev..

[56] Tian J., Ge F., Zhang D., Deng S., Liu X. (2021). Roles of Phosphate Solubilizing Microorganisms from Managing Soil Phosphorus Deficiency to Mediating Biogeochemical P Cycle. Biology.

[57] Bahadir P.S., Liaqat F., Eltem R. (2018). Plant growth promoting properties of phosphate solubilizing Bacillus species isolated from the Aegean Region of Turkey. Turk. J. Bot..

[58] Wang Y.-Y., Li P.-S., Zhang B.-X., Wang Y.-P., Meng J., Gao Y.-F., He X.-M., Hu X.-M. (2020). Identification of phosphate-solubilizing microorganisms and determination of their phosphate-solubilizing activity and growth-promoting capability. BioResources.

[59] Zhu J., Li M., Whelan M. (2018). Phosphorus activators contribute to legacy phosphorus availability in agricultural soils: A review. Sci. Total Environ..

[60] Galavi M., Yosefi K., Ramrodi M., Mousavi S.R. (2011). Effect of bio-phosphate and chemical phosphorus fertilizer accompanied with foliar application of micronutrients on yield, quality and phosphorus and zinc concentration of maize. J. Agric. Sci..

[61] Mącik M., Gryta A., Frąc M., Sparks D.L. (2020). Chapter Two—Biofertilizers in agriculture: An overview on concepts, strategies and effects on soil microorganisms. Advances in Agronomy.

[62] Wyciszkiewicz M., Saeid A., Dobrowolska-Iwanek J., Chojnacka K. (2016). Utilization of microorganisms in the solubilization of low-quality phosphorus raw material. Ecol. Eng..

[63] Ottosen L.M., Kirkelund G.M., Jensen P.E. (2013). Extracting phosphorous from incinerated sewage sludge ash rich in iron or aluminum. Chemosphere.

[64] Severin M., Breuer J., Rex M., Stemann J., Adam C., Van den Weghe H., Kücke M. (2014). Phosphate fertilizer value of heat treated sewage sludge ash. Plant Soil Environ..

[65] Krüger O., Adam C. (2014). Recovery potential of German sewage sludge ash. Waste Manag..

[66] Abis M., Calmano W., Kuchta K. (2018). Innovative technologies for phosphorus recovery from sewage sludge ash. Detritus.

[67] Worwag M. (2018). Recovery of phosphorus as struvite from sewage sludge and sewage sludge ash. Desalination Water Treat..

[68] Vogel T., Kruse J., Siebers N., Nelles M., Eichler-Lobermann B. (2017). Recycled Products from Municipal Wastewater: Composition and Effects on Phosphorus Mobility in a Sandy Soil. J. Environ. Qual..

[69] Römer W., Steingrobe B. (2018). Fertilizer effect of phosphorus recycling products. Sustainability.

[70] Rolewicz M., Rusek P., Borowik K. (2018). Obtaining of granular fertilizers based on ashes from combustion of waste residues and ground bones using phosphorous solubilization by bacteria *Bacillus megaterium*. J. Environ. Manag..

[71] Jastrzębska M., Kostrzewska M.K., Treder K. (2020). Phosphorus Fertilizers From Sewage Sludge Ash and Animal Blood Have No Effect on Earthworms. Agronomy.

[72] FAO (2014). World reference base for soil resources 2014. International Soil Classification System for Naming Soils and Creating Legends for Soil Maps.

[73] Zalba P., Bravo O., Garay M., Peinemann N. (2007). Moisture and temperature effect on soil phosphorus availability. Agrochimica.

[74] Armada E., Roldán A., Azcon R. (2014). Differential Activity of Autochthonous Bacteria in Controlling Drought Stress in Native Lavandula and Salvia Plants Species Under Drought Conditions in Natural Arid Soil. Microb. Ecol..

[75] Natywa M., Selwet M., Maciejewski T. (2014). Effect of some agrotechnical factors on the number and activity soil microorganisms. Fragm. Agron..

[76] Roberts J., Israel D.W. (2017). Influence of Soil Properties and Manure Sources on Phosphorus in Runoff during Simulated Rainfall. Commun. Soil Sci. Plant Anal..

[77] (1997). Soil quality and Determination of pH.

[78] Górecka H., Chojnacka K., Górecki H. (2006). The application of ICP-MS and ICP-OES in determination of micronutrients in wood ashes used as soil conditioners. Talanta.

[79] TIBCO Software Inc. (2017). Statistica (Data Analysis Software System).

[80] Ram H., Malik S.S., Dhaliwal S.S., Kumar B., Singh Y. (2015). Growth and productivity of wheat affected by phosphorus-solubilizing fungi and phosphorus levels. Plant Soil Environ..

[81] Weigand H., Bertau M., Hübner W., Bohndick F., Bruckert A. (2013). RecoPhos: Full-scale fertilizer production from sewage sludge ash. Waste Manag..

[82] Cabeza R., Steingrobe B., Romer W., Claassen N. (2011). Effectiveness of recycled P products as P fertilizers, as evaluated in pot experiments. Nutr. Cycl. Agroecosyst..

[83] Wollmann I., Gauro A., Müller T., Möller K. (2018). Phosphorus bioavailability of sewage sludge-based recycled fertilizers. J. Plant Nutr. Soil Sci..

[84] Thonar C., Lekfeldt J.D.S., Cozzolino V., Kundel D., Kulhánek M., Mosimann C., Neumann G., Piccolo A., Rex M., Symanczik S. (2017). Potential of three microbial bio-effectors to promote maize growth and nutrient acquisition from alternative phosphorous fertilizers in contrasting soils. Chem. Biol. Technol. Agric..

[85] Zhao Y., Mao X., Zhang M., Yang W., Di H.J., Ma L., Liu W., Li B. (2021). The application of *Bacillus megaterium* alters soil microbial community composition, bioavailability of soil phosphorus and potassium, and cucumber growth in the plastic shed system of North China. Agric. Ecosyst. Environ..

[86] Wollmann I., Möller K. (2018). Phosphorus bioavailability of sewage sludge-based recycled fertilizers in an organically managed field experiment. J. Plant Nutr. Soil Sci..

[87] Lekfeldt J.D.S., Rex M., Mercl F., Kulhánek M., Tlustoš P., Magid J., de Neergaard A. (2016). Effect of bioeffectors and recycled P-fertiliser products on the growth of spring wheat. Chem. Biol. Technol. Agric..

[88] Jastrzębska M., Kostrzewska M.K., Saeid A., Treder K., Makowski P., Jastrzębski W.P., Okorski A. (2017). Granulated phosphorus fertilizer made of ash from biomass combustion and bones with addition of* Bacillus megaterium* in the field assessment. Part 1. Impact on yielding and sanitary condition of winter wheat. Przem. Chem..

[89] Raymond N.S., Jensen L.S., van der Bom F., Nicolaisen M.H., Müller-Stöver D. (2019). Fertilising effect of sewage sludge ash inoculated with the phosphate-solubilising fungus Penicillium bilaiae under semi-field conditions. Biol. Fertil. Soils.

[90] Ma P., Rosen C. (2021). Land application of sewage sludge incinerator ash for phosphorus recovery: A review. Chemosphere.

[91] Rymuza K., Turska E., Wielogórska G., Wyrzykowska M., Bombik A. (2012). Evaluation of yield determination of spring wheat grown in monoculture interrupted with stubble crop growth by means of path analysis. Acta Sci. Pol. Agric..

[92] Kuś J., Jośczyk K. (1997). Oddzialywanie wybranych elementow agrotechniki na plonowanie pszenicy ozimej. Fragm. Agron..

[93] Tujaka A. (2007). Krajowy bilans fosforu w ujęciu regionalnym. Studia I Rap. IUNG-PIB.

[94] Balemi T., Negisho K. (2012). Management of soil phosphorus and plant adaptation mechanisms to phosphorus stress for sustainable crop production: A review. J. Soil Sci. Plant Nutr..

[95] Sapek B. (2014). Soil phosphorus accumulation and release–sources, processes, causes. Woda-Śr.-Obsz. Wiej..

[96] Rosyadi I. (2004). Studies on the Agricultural Utilization of Phosphate from Incinerated Sewage Sludge and Meat & Bone Meal.

[97] Zalewska M., Stępień A., Wierzbowska J. (2020). Agronomic evaluation of dried sewage sludge and sewage sludge ash as sources of nutrients for maize. J. Elem..

[98] Militaru B.A., Pode R., Lupa L., Schmidt W., Tekle-Röttering A., Kazamer N. (2020). Using Sewage Sludge Ash as an Efficient Adsorbent for Pb (II) and Cu (II) in Single and Binary Systems. Molecules.

[99] Jastrzębska M., Kostrzewska M.K., Makowski P., Treder K., Jastrzbski W.P. (2017). Granulated phosphorus fertilizer made of ash from biomass combustion and bones with addition of *Bacillus megaterium* in the field assessment. Part 3. Impact on selected properties of soil environment of winter wheat. Przem. Chem..

[100] Jastrzȩbska M., Kostrzewska M.K. (2019). Using an environment-friendly fertiliser from sewage sludge ash with the addition of *Bacillus megaterium*. Minerals.

[101] Vary P.S., Biedendieck R., Fuerch T., Meinhardt F., Rohde M., Deckwer W.D., Jahn D. (2007). *Bacillus megaterium*-from simple soil bacterium to industrial protein production host. Appl. Microbiol. Biotechnol..

[102] Václavková Š., Šyc M., Moško J., Pohořelý M., Svoboda K. (2018). Fertilizer and Soil Solubility of Secondary P Sources—The Estimation of Their Applicability to Agricultural Soils. Environ. Sci. Technol..

[103] Gomathy M., Thangaraju M., Gunasekaran S., Gopal N.O. (2007). Sporulation and regeneration efficiency of phosphobacteria (*Bacillus megaterium* var phosphaticum). Indian J. Microbiol..

[104] Jastrzębska M., Kostrzewska M.K., Makowski P., Treder K., Marks M. (2015). Effects of ash and bone phosphorus biofertilizers on *Bacillus megaterium* counts and select biological and physical soil properties. Pol. J. Environ. Stud..

[105] Murdock L., Call D. (2006). Managing Seasonal Fluctuations of Soil Tests.

[106] Hinsinger P., Plassard C., Tang C., Jaillard B. (2003). Origins of root-mediated pH changes in the rhizosphere and their responses to environmental constraints: A review. Plant Soil.

[107] Bierman P.M., Rosen C.J., Bloom P.R., Nater E.A. (1995). Soil Solution Chemistry of Sewage-Sludge Incinerator Ash and Phosphate Fertilizer Amended Soil. J. Environ. Qual..

[108] Oenema O., Kros H., de Vries W. (2003). Approaches and uncertainties in nutrient budgets: Implications for nutrient management and environmental policies. Eur. J. Agron..

[109] EC (2018). Final Implementation Report for Directive 86/278/EEC on Sewage Sludge: 2013—2015.

[110] Chen W., Chang A.C., Wu L. (2007). Assessing long-term environmental risks of trace elements in phosphate fertilizers. Ecotoxicol. Environ. Saf..

[111] Belhaj D., Elloumi N., Jerbi B., Zouari M., Abdallah F.B., Ayadi H., Kallel M. (2016). Effects of sewage sludge fertilizer on heavy metal accumulation and consequent responses of sunflower (Helianthus annuus). Environ. Sci. Pollut. Res..

[112] Jiao W., Chen W., Chang A.C., Page A.L. (2012). Environmental risks of trace elements associated with long-term phosphate fertilizers applications: A review. Environ. Pollut..

[113] Uprety D., Hejcman M., Száková J., Kunzová E., Tlustoš P. (2009). Concentration of trace elements in arable soil after long-term application of organic and inorganic fertilizers. Nutr. Cycl. Agroecosyst..

[114] Jastrzȩbska M., Saeid A., Kostrzewska M.K., Basladyńska S. (2018). New phosphorus biofertilizers from renewable raw materials in the aspect of cadmium and lead contents in soil and plants. Open Chem..

[115] (1996). Determination of Available Phosphorus in Mineral Soils.

[116] Jalali M., Jalali M. (2020). Effect of organic and inorganic phosphorus fertilizers on phosphorus availability and its leaching over incubation time. Environ. Sci. Pollut. Res..

[117] Tao G.-C., Tian S.-J., Cai M.-Y., Xie G.-H. (2008). Phosphate-solubilizing and -mineralizing abilities of bacteria isolated from soils. Pedosphere.

[118] Qian Ting Y.E.J.-r. (2020). The Mechanism of Dissolving Inorganic Phosphorus by *Bacillus megaterium* ZS-3 and Its Growth Promotion of Cinnamomum camphora. Biotechnol. Bull..

[119] Sárdi K., Naeem M., Ansari A.A., Gill S.S. (2017). Short-Term Transformation and Dynamics of Main Nutrients in Soil. Essential Plant Nutrients: Uptake, Use Efficiency, and Management.

[120] Costa P.B.d., Granada C.E., Ambrosini A., Moreira F., de Souza R., dos Passos J.F.M., Arruda L., Passaglia L.M.P. (2014). A Model to Explain Plant Growth Promotion Traits: A Multivariate Analysis of 2211 Bacterial Isolates. PLoS ONE.

[121] Wolińska A. (2010). Dehydrogenase activity of soil microorganism and oxygen availability during reoxidation process of selected mineral soils from Poland. Acta Agrophys..

[122] Delcǎ E., Stere I. (2013). Influence of chemical fertilizers and biofertilizers on the dynamics of some microbial groups (heterotrophic bacteria, free nitrogen-fixing bacteria) in Chernozem soil of dobrogea (Cumpǎna, valu lui traian). Rom. Agric. Res..

[123] Guan G., Tu S., Li H., Yang J., Zhang J., Wen S., Yang L. (2013). Phosphorus fertilization modes affect crop yield, nutrient uptake, and soil biological properties in the rice-wheat cropping system. Soil Sci. Soc. Am. J..

[124] da Silva F.B.V., do Nascimento C.W.A., Araújo P.R.M. (2017). Environmental risk of trace elements in P-containing fertilizers marketed in Brazil. J. Soil Sci. Plant Nutr..

[125] Sabir M.S., Shahzadi F., Ali F., Shakeela Q., Niaz Z., Ahmed S. (2021). Comparative Effect of Fertilization Practices on Soil Microbial Diversity and Activity: An Overview. Curr. Microbiol..

[126] Li Y., Tremblay J., Bainard L.D., Cade-Menun B., Hamel C. (2020). Long-term effects of nitrogen and phosphorus fertilization on soil microbial community structure and function under continuous wheat production. Env. Microbiol..

[127] Sruthilaxmi C.B., Babu S. (2017). Microbial bio-inoculants in Indian agriculture: Ecological perspectives for a more optimized use. Agric. Ecosyst. Environ..

[128] Gadhave K.R., Devlin P.F., Ebertz A., Ross A., Gange A.C. (2018). Soil Inoculation with Bacillus spp. Modifies Root Endophytic Bacterial Diversity, Evenness, and Community Composition in a Context-Specific Manner. Microb. Ecol..

[129] Ali A.F., Salim H.A., Alsaady M.H.M. (2019). Response of two wheat cultivars to inoculation of Bacillus subtilis and Phosphorus fertilizer. J. Phys. Conf. Ser..

[130] Grzebisz W., Potarzycki J., Biber M., Szczepaniak W. (2003). Reakcja roślin uprawnych na nawożenie fosforem [Response of agricultural crops to nitrogen fertilization]. J. Elem..

[131] Jastrzębska M., Kostrzewska M., Treder K., Jastrzębski W., Makowski P. (2016). Phosphorus biofertilizers from ash and bones—Agronomic evaluation of functional properties. J. Agric. Sci..

[132] Neina D. (2019). The Role of Soil pH in Plant Nutrition and Soil Remediation. Appl. Environ. Soil Sci..

[133] Kucharczak-Moryl E., Moryl A., Żmuda R. (2014). Influence of the environment on the content of arsenic in cultivated soils in Zgorzelec-Bogatynia region. Inż. Ekol..

[134] Czarnowska K. (1996). Total content of heavy metals in parent rocks as reference background levels of soils. Rocz. Glebozn..

[135] Kabata-Pendias A., Pendias H. (1999). Biogeochemistry of Trace Elements.

[136] ME-PL (2016). Ordinance by the Minister of the Environment (Poland) of 1 September 2016 on assessment procedures for the land surface pollution. J. Laws.

[137] IUNG (2017). The Monitoring of the Chemistry of the Polish Arable Soils.

