# Supplementary material for: Phosphorus Fertilizers from Sewage Sludge Ash and Animal Blood as an Example of Biobased Environment-Friendly Agrochemicals: Findings from Field Experiments
Source: Molecules. 2022 Apr 26;27(9):2769. doi: 10.3390/molecules27092769 (PMC9100326; doi:10.3390/molecules27092769)
Supplement: Supplementary file 1 [file molecules-27-02769-s001.zip › molecules-1633962-supplementary.pdf]

Phosphorus Fertilizers from Sewage Sludge Ash and Animal Blood as an Example of Biobased Environment-Friendly Agrochemicals: Findings from Field Experiments

Magdalena Jastrzębska [1,*], Marta K. Kostrzewska [1] and Agnieszka Saeid

[1] Department of Agroecosystems and Horticulture, Faculty of Agriculture and Forestry, University of Warmia and Mazury in Olsztyn, Plac Łódzki 3, 10-718 Olsztyn, Poland; marta.kostrzewska@uwm.edu.pl (M.K.K.)

[2] Department of Engineering and Technology of Chemical Processes, Faculty of Chemistry, Wroclaw University of Science and Technology, Wyspiańskiego 42, 50-376 Wrocław, Poland; agnieszka.saeid@pwr.edu.pl (A.S.)

* Correspondence: jama@uwm.edu.pl (M.J.)

Supplementary information

Table S1. Basic agricultural information for the experiments.

Item	Experiment		
	SW-2016	SW-2017	WW-2017
Wheat cultivar	Monsun	Monsun	Julius
Previous crop	winter rape	spring wheat	winter wheat
Soil tillage system	plough tillage	plough tillage	plough tillage
Fertilization			
K, kg ha^{-1}	83 [1] potassium chloride	83 [1] potassium chloride	83 [1] potassium chloride
N, kg ha^{-1}	130 (60[1] + 50[3] + 20[4]) ammonium sulphate	110 (60[1] + 50[3]) ammonium sulphate	150 (80[2] + 50[3] + 20[4]) ammonium nitrate [2] ammonium sulphate [3,4]
Plant protection			
–Herbicides	florasulam + 2,4-D (29 May)	florasulam + 2,4-D (22 May)	florasulam + 2,4-D (13 May)
–Fungicides	thiophanate-methyl + tetraconazole (9 June) azoxystrobin (8 July)	thiophanate-methyl + tetraconazole (6 June) azoxystrobin + (propiconazole + cyproconazole) (28 June)	fenpropimorph + epoxiconazole + metrafenone (16 May) fluxapyroxad + pyraclostrobin + epoxiconazole (8 June)
–Insecticides	lambda-cyhalothrin (6 June)	deltamethrin (6 June)	deltamethrin (6 June)
–Growth regulators	–	–	trinexapac-ethyl (16 May)
Sowing date	21 April 2016	20 April 2017	4 October 2016
Harvest date	12 August 2016	18 August 2017	4 August 2017

[1] pre-sowing, [2] at post-winter vegetation start, [3] at wheat stem elongation, [4] at wheat heading; – not applied.

Table S2. Atmospheric precipitation and air temperature during the study period according to the Meteorological Station in Bałcyny, Poland.

Year	Days	Month											
		**I**	**II**	**III**	**IV**	**V**	**VI**	**VII**	**VIII**	**IX**	**X**	**XI**	**XII**
**Atmospheric precipitation, mm**													
2016	1-10			0.6	4.8	7.5	12.1	39.6	54.5	7.5	65.1	39.9	41.4
	11-20			7.0	19.2	53.7	28.1	34.0	10.4	1.2	9.2	27.7	19.2
	21-31			12.9	9.1	9.6	26.1	65.0	7.0	8.4	22.0	10.6	17.2
	Total			20.5	33.1	70.8	66.3	138.6	71.9	17.1	96.3	78.2	77.8
2017	1-10	8.2	3.8	24.3	7.7	27.5	16.6	29.4	31.6				
	11-20	7.5	14.0	18.6	30.8	2.3	29.7	20.7	11.7				
	21-31	0.1	22.7	10.1	13.6	4.2	63.6	56.0	11.5				
	Total	15.8	40.5	53.0	52.1	34.0	109.9	106.1	54.8				
1981-2010	Total	30.1	23.1	30.7	29.8	62.3	72.9	81.2	70.6	56.2	51.2	46.1	42.6
Air temperature, °C													
2016	1-10			2.9	10.3	14.1	16.4	17.5	17.7	17.5	9.0	2.7	1.9
	11-20			2.2	9.1	11.8	16.3	18.1	15.6	15.1	5.7	2.7	-0.3
	21-31			5.5	6.9	18.5	21.3	19.9	19.3	11.7	6.1	2.1	1.4
	Average			3.6	8.8	14.9	18.0	18.5	17.6	14.7	6.9	2.5	1.0
2017	1-10	-6.0	-4.3	3.7	9.8	8.5	15.5	16.0	20.8				
	11-20	-2.2	-2.2	4.3	4.3	14.9	17.4	17.2	19.2				
	21-31	-1.6	3.5	7.0	6.1	15.7	17.2	18.5	16.3				
	Average	-3.2	-1.2	5.1	6.7	13.1	16.7	17.3	18.7				
1981-2010	Average	-2.4	-1.6	1.8	7.7	13.2	15.8	18.3	17.7	13.0	8.1	2.8	-1.0

Table S3. Microbiological culture media composition.

Culture medium	Components	Concentration, $g\ L^{-1}$	pH
Tryptic Soy Agar (TSA)	trypticase peptone	15.0	7.3-7.5
	papaic digest of soyabean meal	5.0	
	NaCl	5.0	
	agar	15.0	
Rose-Bengal Chloramphenicol (RBC)	mycological peptone	5.0	7.2
	glucose	10.0	
	KH_2PO_4	1.0	
	$MgSO_4$	0.5	
	rose bengal	0.05	
	chloramphenicol	0.1	
	agar	15.5	

Discussion on seasonal changes in soil pH in the experiments

In experiment SW-2017, the decrease in pH at wheat tillering was likely caused by root activity in response to insufficient available P in the soil. Despite the initial soil pH indicating high availability of P compounds, at this phase of high plant demand for P [1], the rate of its release from the soil pool and from fertilizers seems to have been too slow under conditions of scanty precipitation occurring at that time (11-31 May)(Table S2). Under such circumstances, plant roots exude organic acids that increase

the solubility of P bonded to aluminum (Al), iron (Fe) and manganese (Mn) oxides [2]. In earlier studies by Jastrzębska et al. [3], when weather conditions were more favorable, lowering of soil pH at the wheat early development stages due to plant root activity occurred under no P treatments and after the application of P in the form of phosphorite and SSA-water solution, i.e. less soluble compounds. In turn, the increase in soil pH noted after wheat harvest was probably mostly related to the intensive plant uptake of anions, mainly nitrates (NO_3^-), during vegetation followed by the release of bicarbonate (HCO_3^-) or hydroxyl ions (OH^-) to maintain electrical neutrality at the soil-root interface [4,5].

In experiment WW-2017, the increase in soil pH at winter wheat tillering (spring) was most probably caused by the first part of the N dose applied a little earlier (at post-winter vegetation start) in the form of ammonium nitrate mixed with dolomite meal. A substantial amount of N (80 kg ha^{-1}; see Table 3) was also accompanied by a correspondingly large amount of calcium (Ca). After wheat harvest, there were no more significant changes in soil pH compared to the previous analysis term, indicating a balancing of biogeochemical processes affecting this soil property [5].

Table S4. Reference values for soil PTE content, mg kg^{-1} of soil DM.

Content		As	Cd	Cr	Ni	Pb	Reference
Geochemical background for Poland		2–13	0.03–1.00	2.0–64	0.5–28.5	5.0–59.0	[6,7]
In surface level of Polish soils		0.6–35	0.01–1.6	5–100	0.5–60	5–85	[8]
Permissible in Poland in arable land		10	2	150	100	100	[9]
Monitoring of arable soils	– Poland	0.73–20.7	0.02–68.0	2.4–49.1	1.0–71.1	4.5–857	[10]
	– region	1.52–3.87	0.07–0.18	4.5–30.6	2.6–29.8	7.6–14.4	

References

1. Grzebisz, W.; Potarzycki, J.; Biber, M.; Szczepaniak, W. Reakcja roślin uprawnych na nawożenie fosforem [Response of agricultural crops to nitrogen fertilization]. *J. Elem.* **2003**, *8*, 83-93.
2. Balemi, T.; Negisho, K. Management of soil phosphorus and plant adaptation mechanisms to phosphorus stress for sustainable crop production: A review. *J. Soil Sci. Plant Nutr.* **2012**, *12*, 547-561.
3. Jastrzębska, M.; Kostrzewska, M.; Treder, K.; Jastrzębski, W.; Makowski, P. Phosphorus biofertilizers from ash and bones—agronomic evaluation of functional properties. *J. Agric. Sci.* **2016**, *8*, 58-70, doi:10.5539/jas.v8n6p58.
4. Hinsinger, P.; Plassard, C.; Tang, C.; Jaillard, B. Origins of root-mediated pH changes in the rhizosphere and their responses to environmental constraints: a review. *Plant Soil* **2003**, *248*, 43-59, doi:10.1023/A:1022371130939.
5. Neina, D. The Role of Soil pH in Plant Nutrition and Soil Remediation. *Appl. Environ. Soil Sci.* **2019**, *2019*, 5794869, doi:10.1155/2019/5794869.
6. Kucharczak-Moryl, E.; Moryl, A.; Żmuda, R. Influence of the environment on the content of arsenic in cultivated soils in Zgorzelec-Bogatynia region. *Inż. Ekol.* **2014**, *2014*, 107-116.
7. Czarnowska, K. Total content of heavy metals in parent rocks as reference background levels of soils. *Rocz. Glebozn.* **1996**, *47*, 43-50.
8. Kabata-Pendias, A.; Pendias, H. *Biogeochemistry of trace elements*; Polish Scientific Publishing Company: Warsaw, Poland, 1999.
9. ME-PL. Ordinance by the Minister of the Environment (Poland) of 1 September 2016 on assessment procedures for the land surface pollution. *J. Laws* **2016**, *2016*, 1395.
10. IUNG. *The monitoring of the chemistry of the Polish arable soils*; Institute of Soil Science and Plant Cultivation: Puławy, Poland, 2017.
